# Ideal body weight in the precision era: recommendations for prescribing in obesity require thought for computer-assisted methods

**DOI:** 10.1136/archdischild-2019-318370

**Published:** 2019-12-12

**Authors:** Nicholas Appelbaum, Carmen Rodriguez-Gonzalvez, Jonathan Clarke

**Affiliations:** 1 Surgery and Cancer, Imperial College London, London, UK; 2 Helix Centre for Design in Healthcare, Imperial College London, London, United Kingdom

**Keywords:** intensive care, nursing

As the prevalence of childhood obesity continues to increase, there has been comparatively slow growth in the literature describing how best to dose obese children. For medications with low lipid solubility where doses are calculated by the total body weight (TBW) of the child, increasing adiposity may lead to the administration of doses well in excess of that required for therapeutic effect, and potentially beyond the safe therapeutic interval of the medication. This concern underlies recommendations to use alternative bodyweight measurements for some medications when dosing obese children.

There are numerous alternative bodyweight scalars which are used in obese children, including ideal body weight (IBW), lean body mass and adjusted body weight.^1^ IBW is probably the most commonly used in obesity and is the only alternative to TBW mentioned in the British National Formulary for Children.^2^ There is little consensus on how best to calculate IBW however, and we have previously shown that there is significant discrepancy in the proportion of TBW represented by IBW depending on which of five published methods (Traub, Moore, body mass index (BMI) method, American Dietetic Association, McLaren) is chosen.^3^ A 2016 study by Collier *et al* 4 found that there was a generally poor understanding of when and how to calculate IBW in a cohort of UK-based paediatricians.

Notwithstanding variability in IBW according to the method chosen to calculate it and applying this method correctly, there remains an additional challenge: as children increase in BMI, when should we apply IBW over TBW? It is well known that increased cognitive burden contributes to medication error, and using more than one weight scalar simultaneously clearly increases complexity. The movement towards electronic prescribing with decision support, however, arguably makes complex calculations less of a problem, but the literature is not clear when IBW should be applied in this context. In the UK, one of the most comprehensive pieces of guidance was published by UK Medicines Information with input from the Neonatal and Paediatric Pharmacists Group in 2018,^1^ where it was recommended that TBW be used for all doses until the child is clinically obese, that is, above the 98th percentile of BMI-for-age, after which an appropriate weight scalar should be chosen if there is a recommendation to use one.

**Figure 1:**
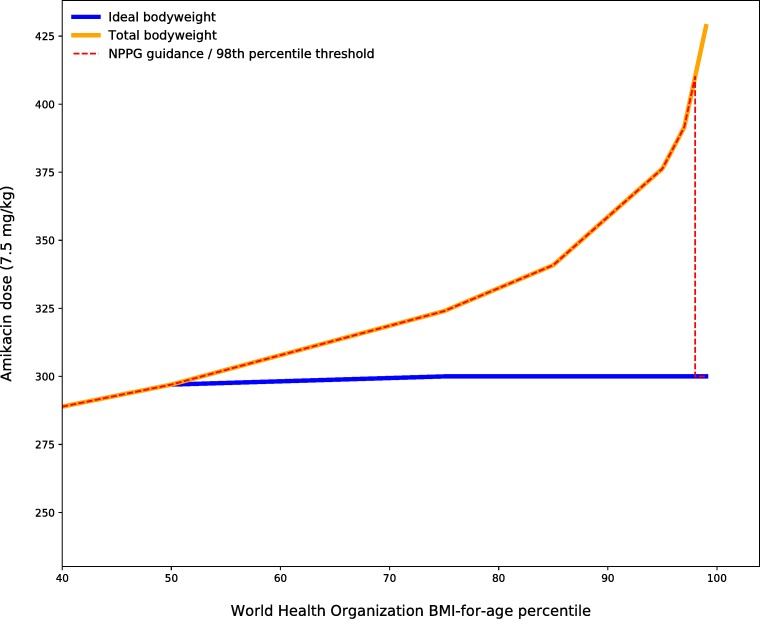
Amikacin dosed by BMI centile for a 12-year-old boy (1.5 m) using total body weight, ideal body weight and ideal body weight only in clinical obesity. BMI, body mass index; NPPG, Neonatal and Paediatric Pharmacists Group.

Following this guidance, for a 1.5 m tall, 12-year-old boy on the 97th centile by BMI, the dose of amikacin (7.5 mg/kg) recommended based on dosing by TBW would be 392 mg. Paradoxically, according to this guidance, a child on the 98th centile would receive a dose calculated using IBW (BMI method) of 300 mg (23% lower), despite having arguably the same pharmacokinetic considerations. The exponential nature of the discrepancy between IBW and TBW dosing for increasing BMI is shown in figure 1. While this cut-off may seem logical where pragmatism trumps complexity in non-computerised workflows, for computer-implemented methods it makes little sense. As the paediatric obesity epidemic continues, electronic prescribing with decision support brings the tools with which to safely account for extremes of weight. The guidance according to which these emerging tools are to be configured, however, lags sorely behind.
